# Influence of Parental Lifestyle and Dietary Patterns on Mediterranean Diet Adherence in Children and Adolescents: A Cross-Sectional Study

**DOI:** 10.3390/nu18101576

**Published:** 2026-05-15

**Authors:** Sevasti Peraki, Izolde Bouloukaki, Antonios Christodoulakis, Dimitrios Vavoulas, Ioanna Tsiligianni

**Affiliations:** 1 Department of Social Medicine, School of Medicine, University of Crete, 71500 Heraklion, Greece; sevastiperaki@gmail.com (S.P.); i.bouloukaki@uoc.gr (I.B.); dk.vavoulas@gmail.com (D.V.); i.tsiligianni@uoc.gr (I.T.); 2 Department of Primary Care and Population Health, Medical School, University of Nicosia, 2417 Nicosia, Cyprus

**Keywords:** lifestyle behaviors, health behaviors, dietary patterns, Mediterranean diet, family environment, parents, pediatric nutrition, adolescents, KIDMED, child obesity

## Abstract

**Background/Objectives**: Adherence to the Mediterranean diet (MD) is associated with reduced risk of non-communicable diseases but has declined among children, even in traditionally high-adherence settings such as Greece. As parental lifestyle behaviors strongly influence children’s dietary patterns, this study examined the associations between parental lifestyle factors and children’s MD adherence in Crete, Greece. **Methods**: A total of 760 parent–child dyads participated in this cross-sectional study. Children’s adherence to the MD was assessed using the KIDMED index. Parents completed validated instruments, including the MEDAS (MD adherence), IPAQ (physical activity), PSQI (sleep quality), and NLS (nutrition literacy), along with questions on dietary habits and screen time behaviors. ANOVA/Kruskal–Wallis tests and multivariable linear regression identified predictors of KIDMED scores. **Results**: Mean KIDMED score was 5.95 ± 2.65; 32% achieved optimal adherence. Younger children showed higher adherence. Higher children’s adherence to MD was positively associated with parental MD adherence (β = 0.493), urban residence (β = 0.544), higher parental education (β = 0.493), consistent daily meal routines (breakfast and mid-morning and mid-afternoon snacks), higher water intake, and fresh juice consumption (all *p* < 0.05) were positively associated with parental MD adherence. Conversely, lower adherence was associated with parental age ≥ 45 years (β = 0.987), higher parental BMI (β = 0.072), consumption of sugar-sweetened (β = 0.390) or artificially sweetened beverages (β = 0.497), and weekend screen time ≥ 3 h/day (β = 0.383) (all *p* < 0.05). **Conclusions**: Children’s adherence to the MD is strongly associated with parental dietary behaviors and structured meal routines. These findings support family-focused interventions that emphasize parental dietary role modeling to counter declining MD adherence among Mediterranean youth.

## 1. Introduction

Non-communicable diseases (NCDs), including cardiovascular disease, type 2 diabetes (T2D), and cancer, remain the leading cause of mortality worldwide [1,2,3]. In 2021, more than 17 million people under the age of 70 died from NCDs, with approximately 11 million of these deaths attributed to modifiable lifestyle behaviors [3]. These behaviors are often established during childhood and tend to persist into adulthood, shaping lifelong consequences that substantially increase future NCD risk [4]. Among these lifestyle behaviors, unhealthy dietary patterns and insufficient physical activity are consistently identified as major contributors to NCD development [5]. Emerging evidence also highlights the important role of sleep, both in shaping eating behaviors and in influencing NCD risk [6]. Together, these behaviors often contribute to excess body fat (manifesting as overweight and obesity), which constitutes a major metabolic risk factor underlying many NCDs across all ages [1], especially in children and adolescents [7].

Childhood obesity has risen sharply over the past four decades, with most countries reporting persistently high and increasing rates of overweight and obesity [8]. This trend mirrors patterns seen in adult populations, and obesity frequently persists into adulthood due to continued exposure to obesogenic environments and the maintenance of unhealthy behaviors established early in life. Prolonged early-life adiposity has been associated with the earlier onset of chronic diseases and greater morbidity in young adulthood [9,10]. This growing public health challenge is especially pronounced in Mediterranean countries, where childhood obesity rates remain among the highest in Europe [11,12]. In Greece, the prevalence is particularly alarming, with 37.5% of children aged 2–14 years living with overweight or obesity—the highest reported rate in the European Union [13].

In light of these findings, promoting healthy dietary patterns during childhood is essential. The Mediterranean diet (MD), in particular, has consistently been shown to enhance diet quality and reduce obesity risk in children [14]. Characterized by a high intake of fruits, vegetables, whole grains, legumes, fish, and olive oil, the MD offers a nutrient-dense, antioxidant-rich profile [15,16], which is associated with lower cardiometabolic risk and protection against chronic disease [15,17,18]. Children adhering closely to MD tend to have lower body mass index (BMI) and reduced risk of overweight and obesity [19]. Despite these benefits, adherence to the MD has declined across Mediterranean regions, largely due to greater consumption of ultra-processed and nutrient-poor foods [20,21]. Parents play a central role in this shift, as their own eating habits, food choices, and broader lifestyle behaviors strongly influence the dietary patterns their children adopt [22].

Beyond behavioral modeling, parents shape children’s eating habits through the foods available in the home, family meal routines, and related behaviors, such as physical activity, sleep, and screen use [23,24]. Evidence consistently shows that children are more likely to adopt healthier dietary behaviors when their parents engage in them as well [25]. The social learning theory suggests that children learn behaviors by observing and mimicking influential people around them, primarily their parents [26]. This means that when it comes to diet, children are likely to copy/mimic their parents’ food choices, eating habits, and drink preferences. However, these behaviors can also be shaped by another significant but less explored parental factor, namely nutrition literacy, which is the ability to find, understand, and use nutritional information for daily decisions [27]. Although higher nutrition literacy has been associated with better personal eating choices in some cases [28], the link between parents’ nutrition literacy and their children’s eating behaviors remains unclear. Studies in Greece indicate that while parental nutrition literacy is generally good, it does not always lead to ideal dietary habits, suggesting that environmental, socioeconomic, and structural challenges create a wider gap between knowledge and behaviors [29]. Therefore, it is crucial for public health to determine if parental nutrition literacy independently influences children’s adherence to the MD.

Studies have examined how parental characteristics influence children’s eating behaviors [24,30,31,32,33,34,35], but very few have focused specifically on the relationship between parents’ adherence to the MD and the corresponding adherence of their children [36,37]. Furthermore, there is a lack of evidence examining how multiple parental lifestyle behaviors—such as diet quality, physical activity, sleep quality, and nutrition literacy—collectively influence children’s adherence to the MD. This gap is particularly evident in Greece, especially Crete. This island’s traditional diet was the linchpin for the development of the MD concept, according to the Seven Countries Study [38]. Therefore, the aim of this cross-sectional study was to investigate the associations between parental lifestyle behaviors, including adherence to the MD, physical activity, sleep quality, and nutrition literacy, and children’s adherence to the MD in Crete, Greece.

## 2. Materials and Methods

### 2.1. Study Design

This cross-sectional study was conducted between December 2025 and March 2026, following the STROBE guideline for observational studies [39]. Participants were recruited through convenience sampling by disseminating an anonymous online survey via social media platforms, parent networks, and local schools across the Mediterranean region of Crete, Greece. An email invitation was sent to all kindergartens, primary and secondary schools in Crete, providing information about the study and a link to the online questionnaire created in the online platform “Google Forms”.

School representatives distributed the questionnaire to parents via email. Through this sampling method, we aimed to ensure representation across the four prefectures of the Region of Crete (Heraklion, Chania, Rethymno, and Lasithi), including both rural and urban areas. The survey required approximately 20 min to complete. Parents (mother or father) completed the questionnaire on behalf of themselves and their child. Prior to participation, informed consent was obtained from all participants. A completed STROBE checklist can be found in the Supplementary Material Table S1.

### 2.2. Study Population

Eligible participants were required to (i) be a parent of at least one child ≤ 18 years, (ii) have internet access to complete the online survey, (iii) have sufficient Greek language proficiency to accurately complete the survey, and (iv) provide informed consent. Individuals who did not meet these criteria did not participate in the study, and no additional exclusion criteria were applied.

### 2.3. Sample Size

A sample size calculation was conducted using G*Power software (version 3.1) for the most complex analysis planned in this study, multivariate linear regression, examining the relationship between children’s MD adherence and parental lifestyle behaviors [40,41]. For a medium effect size (f^2^ = 0.15), an alpha level of 0.05, and a desired power of 0.95, the analysis indicated that a minimum of 90 participants would be required. Taking into account a potential 20% attrition rate and considering that the most complex analysis requires a sample size of 90 participants, the minimum target sample size was set at 112 participants. As a result, the 760 participants were more than adequate for our study.

### 2.4. Data Collection

Data were collected through an anonymous online survey consisting of eight questions. These included, a demographic questionnaire for the parents developed for this study (assessing biological sex, age, height, current weight, area of residence, marital and employment status, education level, smoking status, number of children and number of non-adult children), the Greek version of the MD Quality Index in Children and Adolescents, the Greek version of the MD Adherence Screener, the Greek version of the International Physical Activity Questionnaire, the Greek version of the Pittsburgh Sleep Quality Index, the Greek version of the Nutrition Literacy Scale, questions on dietary habits (water consumption, intake of sugar-sweetened beverages, and meal frequency) and questions on screen time (excluding working hours). Parents were also asked to provide their child’s age. Completion time for the full set of questionnaires was approximately 20–30 min.

### 2.5. Instruments

Written permission was obtained for each questionnaire from the original developers as well as from the research teams responsible for the Greek translations and validations.

#### 2.5.1. Mediterranean Diet Adherence Screener (MEDAS)

The Mediterranean Diet Adherence Screener (MEDAS) is a questionnaire that has been used in various settings and has been validated in several different populations worldwide to evaluate the level of adherence to MD [42,43,44,45]. The MEDAS is a dichotomy-type questionnaire and consists of 14 items, requesting participants to report their frequency of main food group consumption based on the MD components (how many times per day/week?) and their dietary habits (do you use/prefer?) related to the MD [46]. In this study, a validated Greek version of MEDAS was used to measure parents’ level of adherence to the MD [46,47]. The evaluation of each query is determined by the degree to which the participants adhere to the MD, with a rating of 1 being assigned if the criteria are met and a rating of 0 being assigned if the criteria are not met [46]. Thus, the total MEDAS scores are divided into thirds and may range from 0 to 14, with higher total scores indicating a good adherence to the MD [46]. Specifically, scores between 1 and 4 indicate poor adherence; 5–7, average adherence; and 8–14, good adherence to the MD [46].

#### 2.5.2. Mediterranean Diet Quality Index in Children and Adolescents (KIDMED)

The Mediterranean Diet Quality Index in Children and Adolescents (KIDMED) describes adherence to the MD pattern in children and adolescents [48]. Previous studies have indicated adequate levels of internal consistency, with it being used in studies in and out of the Mediterranean region, including Greece [49,50,51]. In this study, the validated Greek version of KIDMED was used to explore children’s dietary habits [52]. Participants had to fill out a separate questionnaire for each of their children. This questionnaire is a dichotomy-type questionnaire and consists of 16 questions, answered with a “yes” (positive) or a “no” (negative) [48]. Questions are based on the principles of the MD dietary pattern and the general dietary guidelines for youth [48]. The KIDMED contains two types of queries: four that negatively correlate with a high-quality diet and are assigned a value of −1, and twelve with a positive aspect and are assigned a value of +1 [48]. According to the KIDMED scoring process, the resulting score is divided into thirds and may range from 0 to 12, with higher total scores indicating a good adherence to the MD [48]. Specifically, scores ≤ 3 indicate poor adherence and low diet quality; 4–7 average adherence and relatively sufficient diet quality; and 8–12 optimal adherence to the MD and sufficient dietary habits [48]. For participants who had two or more children, the mean of individual scores was calculated and used in the analysis in order to avoid selection bias.

#### 2.5.3. International Physical Activity Questionnaire (IPAQ)

The International Physical Activity Questionnaire (IPAQ) is an 8-item self-administered instrument designed to monitor physical activity (PA) and inactivity in individuals [53]. A global evaluation of the IPAQ suggested that it exhibits sufficient consistency and adequate validity, comparable to other self-reported PA questionnaires [53]. In this study, the validated Greek version of IPAQ was utilized to determine the levels of PA parents engage in during a usual week [54,55]. More specifically, IPAQ was used to determine vigorous, moderate, and walking PAs during the last week, thereby producing an overall physical activity score (PAscore), expressed in metabolic equivalent of the task (MET)-minutes per week (MET min wk^−1^) [53,56]. Thus, the resulting IPAQ score is classified into three PA status categories: (1) low PA class (total PAscore < 600 MET min wk^−1^); (2) modest PA class (total PAscore ≥ 600 MET min wk^−1^); and (3) great PA class (total PAscore ≥ 3000 MET min wk^−1^) or vigorous (total PAscore ≥ 1500 MET min wk^−1^) [53,56].

#### 2.5.4. Pittsburgh Sleep Quality Index (PSQI)

The Pittsburgh Sleep Quality Index (PSQI) is a self-reported index evaluating sleep quality. PSQI is a standard instrument that has been validated as differentiating poor from good sleep [31,32]. The PSQI is a 19-item self-rated questionnaire that evaluates subjective sleep quality and quantity, sleep habits related to quality, and occurrence of sleep disturbances in adults over the period of the last month. In previous studies, it has been demonstrated that the internal consistency of this questionnaire is adequate (Cronbach’s alpha 0.70–0.83) and that it possesses construct validity in various populations. In this study, we utilized the validated Greek version of the PSQI to evaluate the sleep quality of parents living in Crete, Greece. PSQI consists of 19 items clustered into 7 components: (1) subjective sleep quality (one item); (2) sleep latency (two items); (3) sleep duration (one item); (4) habitual sleep efficiency (three items); (5) sleep disturbances (nine items); (6) use of sleep medication (one item); and (7) daytime dysfunction (two items). Each component score has a range from 0 to 3, where 0 indicates no difficulty and 3 indicates severe difficulty. The final score is generated by summing the scores of all aforementioned items, which vary between 0 and 21, with a higher score indicating a greater negative impact on sleep quality. A total global PSQI score ≥ 5 indicates poor sleep.

#### 2.5.5. Nutrition Literacy Scale (NLS)

The Nutrition Literacy Scale (NLS) is a tool that assesses reading comprehension and measures the ability of an individual to understand nutritional information [57]. NLS has been widely used in various populations [58]. A Greek validated version of NLS was utilized in this study to evaluate nutrition literacy (NL) in parents living in Crete, Greece [59]. NLS consists of 29 items, where in each one, a word is missing. The participant has four options to select from, of which only one is correct, to complete the sentence [57]. The total NLS score is calculated by summing the correct answers, while the range of the total score is 0–29 [57]. The higher the score, the higher the NL. The total NLS score is categorized into three sections: inadequate NL (score of <8), marginal NL (score of 8–15), and adequate NL (score of >15) [57].

#### 2.5.6. Dietary Habits and Screen Time

A set of items assessing dietary habits and screen time of the parents was developed based on previous studies [34,60]. The items were adapted from previously published studies and were used to capture demographic data and specific behaviors descriptively; therefore, no formal validation of this questionnaire as a psychometric scale was undertaken [34]. Nine items focused on dietary behaviors and were organized into two sections: (1) frequency of consumption of water and beverages with sugar or sweeteners (5 items) and (2) frequency of breakfast and snack consumption (4 items). In the first section, response options ranged from 0 to 6, where 0 indicated never, 1 indicated 1 portion per week, 2 indicated 2–4 portions per week, 3 indicated 5–6 portions per week, 4 indicated 1–2 portions per day, 5 indicated 3–4 portions per day, and 6 indicated more than 5 portions per day. In the second section, each item was scored on a 0–7 scale, with 0 indicating never and 7 indicating daily. Screen time (excluding working hours) was assessed using two additional items capturing average hours per day on weekdays and weekends, respectively. Each item was scored on a 0–9 scale, where 0 indicated never, and 9 indicated more than 8 h per day.

### 2.6. Statistical Analysis

We performed the statistical analysis using IBM SPSS version 26.0 (SPSS Inc., Chicago, IL, USA). The Shapiro–Wilk test was applied to assess the normality of continuous variables, supplemented by visual inspection of Q–Q plots and histograms, as well as evaluation of skewness and kurtosis. Variables with a normal distribution are presented as means and standard deviations (SDs), whereas non-normally distributed variables are reported as medians with interquartile ranges (25–75th percentile). Categorical variables are summarized as frequencies and percentages. Differences in parental lifestyle behavior measures across the three KIDMED adherence categories (poor, medium, and optimal) were examined using one-way ANOVA for normally distributed continuous variables and the Kruskal–Wallis test for non-normally distributed variables. The KIDMED score demonstrated close agreement between the mean and median, minimal skewness (−0.162), and negligible kurtosis (0.087), with no evidence of floor or ceiling effects. Visual inspection of histograms and normal Q–Q plots supported approximate normality. Formal tests of normality were statistically significant, as expected in large samples, but were considered of limited practical relevance. Linear regression was therefore deemed appropriate and robust for the primary analyses. Therefore, multivariate linear regression models were used to evaluate the associations between children’s MD adherence (KIDMED score) and parental lifestyle behaviors. Furthermore, multivariate linear regression models were applied to examine associations between parental adherence to the MD (MEDAS score) and the full range of parental characteristics and lifestyle behaviors. Beta coefficients and standard errors (SEs) were estimated, adjusting for potential confounders, including child age, parental age, sex, BMI, educational level, family status, smoking status, and area of residence. Statistical significance was defined as *p* < 0.05 (two-tailed).

## 3. Results

### 3.1. Children’s Characteristics

The final sample consisted of 760 parent–child dyads. Children had a mean age of 9.6 ± 4.4 years, and the mean KIDMED score was 5.95 ± 2.65, indicating overall moderate adherence to the Mediterranean diet (MD). Optimal adherence was observed in 32% of children, while 18% showed poor adherence. Higher MD adherence was consistently observed in younger children (Table 1). Item-level analysis of the KIDMED questionnaire indicated high use of olive oil (98%) and regular consumption of fruits (70% at least once daily; 46% at least twice daily), and legumes (59%), alongside unfavorable patterns such as frequent sweets intake (67%) and breakfast skipping (77%).

### 3.2. Parental Characteristics

Parental characteristics are summarized in Table 1 and Table 2. Most parents were female, resided in urban areas, and had post-secondary education. Overall parental adherence to the MD was high (mean MEDAS score 8.26 ± 1.85), while a substantial proportion reported poor sleep quality (67%). Nearly all parents demonstrated adequate nutrition literacy (99%).

The majority of the parents consumed fresh juice at least weekly (81%), and around half reported drinking at least five portions of water daily (51%). Over half ate breakfast daily (56%), a third had a mid-morning snack (33%), and a similar proportion had a mid-afternoon snack (32%). Additionally, 28% of parents spent three or more hours daily on screen time (time spent using devices with screens, such as smartphones, tablets, computers, TVs, etc.). This screen time score (three or more hours) further increased to 40% of the parents during non-working hours on weekdays (Table 3).

Higher adherence to the MD, as measured by the MEDAS score, was significantly associated with lower BMI (β = −0.064, *p* = 0.006) and better sleep quality, reflected by lower PSQI scores (β = −0.177, *p* < 0.001).

### 3.3. Associations of Parental Characteristics and Behaviors with Children’s Adherence to MD

In unadjusted analyses, parental age ≥ 45 years and higher BMI were associated with lower children’s KIDMED scores, whereas urban residence and higher educational attainment were associated with higher adherence (Table 1). Parental physical activity, sleep quality, and nutrition literacy were not significantly associated with children’s MD adherence (Table 2).

Multiple linear regression analysis revealed several independent predictors of children’s adherence to the Mediterranean Diet (MD) after accounting for all covariates (Table 4). Regarding sociodemographic factors, older children were less likely to adhere to the MD (β = −0.135, *p* < 0.001), as were children of older parents (β = −0.987, *p* < 0.001) and those with higher parental Body Mass Index (BMI) (β = −0.072, *p* < 0.001). Conversely, living in an urban area (β = 0.544, *p* = 0.005) and higher parental educational attainment (β = 0.493, *p* = 0.014) were associated with better adherence.

Parental dietary habits were the strongest influence on children’s MD adherence in our study. Specifically, higher parental adherence to the MD was a significant positive predictor for their children’s adherence to the MD (MEDAS: β = 0.493, *p* < 0.001). Structured daily meal routines also showed a positive association with higher MD adherence, including daily breakfast (β = 1.294, *p* < 0.001), mid-afternoon snacks (β = 1.022, *p* < 0.001), and mid-morning snacks (β = 0.810, *p* < 0.001). Beverage choices, such as weekly fresh juice consumption (β = 0.716, *p* = 0.002) and adequate water intake (β = 0.482, *p* = 0.008), were also positively associated with children’s MD adherence (Table 4). Conversely, unhealthy parental habits were negatively associated with children’s MD adherence. Specifically, weekly consumption of artificially sweetened beverages (β = −0.497, *p* = 0.008) and sugar-sweetened beverages (β = −0.390, *p* = 0.047) was positively associated with lower KIDMED scores, as was excessive weekend screen time (β = −0.383, *p* = 0.044). In contrast, physical activity level, sleep quality, nutrition literacy, consumption of other juice varieties, late-evening snacking, and weekday screen time were not significantly associated with children’s adherence to MD (all *p* > 0.05).

## 4. Discussion

### 4.1. Main Findings

The present study aimed to examine the relationship between parental sociodemographic characteristics, lifestyle behaviors, and children’s adherence to the MD based on parents’ reports in a large sample of parent–child dyads from Crete, Greece. Our results indicate that several parental characteristics, including younger age, higher educational attainment, lower BMI, residence in urban areas, greater adherence to the MD, and healthier daily habits, such as adequate water intake, having regular breakfasts, and snack consumption, were positively associated with higher levels of children’s adherence to the MD. Additionally, children’s adherence was lower when parents reported ≥ 3 h of screen time during non-working hours on weekends. Overall, the findings highlight the potential influence of the family environment on children’s dietary patterns, confirming that parental lifestyle choices-particularly their own adherence to the MD and consistent meal routines, could play a crucial role in shaping children’s nutritional behaviors.

Consistent with evidence showing that children from Mediterranean regions are gradually shifting away from traditional dietary habits [20,21,37], the present study found that only one-third of children exhibited optimal adherence to the MD, while almost half displayed moderate adherence (49%), and 18% had poor adherence. Nevertheless, this proportion of optimal adherence is higher than that reported in other national studies, where estimates range from 4.3% to 22.7% [30,37,61,62].

This finding might be partially due to the study’s location, which was Crete. The Seven Countries Study identified Crete as a region with exceptionally low rates of coronary heart disease globally, a phenomenon linked to the traditional Cretan diet [38]. In Crete, traditional dietary habits may be more strongly preserved than in other parts of Greece. However, this interpretation should be viewed with caution. The MEDIS Study, which examined elderly Mediterranean islanders, including those from Crete, found only moderate overall adherence to the MD, even within this region historically aligned with the MD [63]. Furthermore, a recent study on trends in Spanish children and adolescents reported declining MD adherence over two decades due to reduced intake of fish, legumes, and fruit, alongside increased fast-food consumption [64]. This, unfortunately, along with our findings, suggests a gradual decline in traditional and healthier lifestyle practices across generations [63]. Although some core MD practices remained prevalent (e.g., olive oil use and weekly legume consumption), concerning patterns were also apparent, such as daily sweets consumption (67% of children) and frequent breakfast skipping (77%), reflecting the growing influence of Westernized food habits even in Crete [65,66], underscoring the need for more targeted interventions.

Several parental characteristics were strongly associated with children’s adherence to the MD in our study. Specifically, younger parental age, lower BMI, higher educational attainment, and urban residence predicted better dietary adherence in children. These findings align with previous studies suggesting that younger individuals with lower BMI [36,67] and higher-educated parents [68,69,70,71,72] may be more health-conscious and more receptive to nutrition information, resulting in healthier household food environments. Likewise, the inverse relationship between parental BMI and children’s KIDMED scores from our study may reflect shared familial eating habits, modeling of dietary behaviors, and the transmission of obesogenic lifestyle patterns across generations [37,73]. The positive association observed for urban residence may reflect differences in the local food environment, as urban areas often provide greater availability of diverse food options and nutrition-related resources. Evidence from the DELICIOUS project [67], which examined dietary patterns among children across five Mediterranean countries, also highlights marked urban–rural differences in KIDMED scores, with children living in urban areas showing higher adherence to the MD, suggesting that the living environment may influence adherence to healthier dietary habits. However, these associations are not universally observed. In a Croatian cross-sectional study, which used the same KIDMED–MEDAS pairing as our study, in 2.623 parent–child dyads, parental education was not a significant predictor after adjustment for parental MD adherence [36]. Furthermore, a recent Italian study found that parenting style (specifically, didactic/authoritative) was a stronger predictor of children’s KIDMED scores than parental education alone, suggesting that parenting behaviors may matter more than sociodemographic characteristics themselves [74]. This suggests that the effect of education on children’s diet may be partially explained by parental dietary quality rather than operating independently, a possibility that could not be tested in our cross-sectional design.

Higher parental adherence to the MD (MEDAS score) emerged as one of the stronger predictors of children’s MD adherence, emphasizing the importance of modeling health-promoting behaviors within the family. This strong association between parental and child adherence to the MD in our study is consistent with previous findings showing that parents’ own adherence is one of the most influential predictors of children’s dietary patterns [36,37,75]. Parental meal routines, including daily breakfast, mid-morning snacks, and mid-afternoon snacks, were also positively associated with children’s KIDMED scores. Regular family meal patterns may foster structured eating, reduce the likelihood of unhealthy snacking, and create opportunities for modeling healthy dietary behaviors [34,67,76,77,78,79]. The positive association between parental water and fresh juice consumption and children’s adherence further supports the role of shared household dietary practices. However, the positive association between parental fresh juice consumption and children’s Mediterranean Diet adherence should be interpreted cautiously. Fresh juice is not a core component of the Mediterranean diet and is high in free sugars. Thus, this finding could reflect a broader pattern of lifestyle or act as a proxy for socioeconomic status rather than an independent beneficial dietary factor. Parental dietary habits are likely to play a key role in shaping children’s eating behaviors, both through role-modeling and by regulating the types of foods available at home. Within this framework, evidence from various study designs shows that when healthy foods are accessible and unhealthy options are limited, children are more likely to consume healthier items [80,81,82]. These findings align with Social Learning Theory, which suggests that children acquire behaviors through repeated observation of and exposure to role models, primarily parents [26]. In the present study, the consistent associations between parental MD adherence, structured meal routines, and beverage choices with children’s KIDMED scores suggest that the home dietary environment functions as the primary context for this observational learning, where parental food behaviors are not merely modeled but normalized through daily routine. This is further supported by the Theory of Planned Behavior, whereby parents who perceive healthy eating as a personal and social norm are more likely to create home environments that reinforce those same norms in their children [83]. Collectively, these theories and our findings highlight the potential pivotal role of the family environment as one of the main contributors to dietary habits in children and adolescents. Meanwhile, parental intake of sugar-sweetened and artificially sweetened beverages was linked to lower child MD adherence, indicating that consumption of calorically dense, nutrient-poor beverages within the home may promote similar habits in children [84,85]. Previous studies indicate that parents significantly influence children’s eating behaviors, with stronger parent–child associations reported for patterns of intake of sweets, fats, and fast food further linked to parents’ own fast-food consumption habits [86,87,88]. Notably, parental weekend screen time ≥3 h was also negatively associated with children’s dietary adherence, suggesting that lifestyle routines beyond nutrition-increased screen time with associated sedentary behaviors-may cluster within families, potentially influencing eating patterns through reduced structure, distraction eating, or increased exposure to unhealthy food marketing. Although existing studies primarily examine children’s screen time, evidence consistently shows that increased screen use among children is associated with poorer dietary habits and lower KIDMED scores [89,90,91].

Contrary to expectations, parental physical activity levels, sleep quality, and nutrition literacy were not directly associated with children’s adherence to the MD. This may partly reflect the characteristics of our sample, as most parents reported high physical activity levels and nearly universal adequate nutrition literacy, limiting variability and reducing the likelihood of detecting significant associations. The results for nutritional literacy are consistent with previous research in Greece, where parental nutrition literacy is generally high, yet adherence to the MD remains suboptimal [92,93,94]. This highlights a widespread knowledge-behavior gap: that having sufficient nutritional knowledge does not automatically lead to healthier eating habits [27]. Furthermore, a systematic review indicated a varied link between nutrition literacy and diet quality, with many studies finding no significant correlation [95]. Factors like structural, economic, time, and cultural obstacles, along with habitual and/or emotionally influenced food decisions, often outweigh an individual’s nutritional knowledge in shaping eating habits [96]. Additionally, while nutrition literacy programs designed for specific groups have shown some promise in improving dietary outcomes, their impact in real-world family settings is still limited [97]. This suggests that in populations with high nutrition literacy, like ours, adherence to the MD is potentially more significantly influenced by observed behaviors, the food environment within the home, and structural factors, rather than just nutrition knowledge [98]. Furthermore, while other studies report that only about half of parents engage in sufficient physical activity [62,99], the higher levels observed in our population may have reduced the ability to detect meaningful associations with children’s dietary adherence. Meanwhile, the MEDIS Study, which included Cretan populations, also found moderate overall MD adherence among elderly Mediterranean islanders, highlighting that even in regions where the MD originated, adherence varies, and lifestyle factors [63,100].

Notably, only parental sleep quality was positively associated with parents’ own adherence to the MD, which in turn was strongly related to higher MD adherence in their children. Poorer sleep quality was associated with lower parental MD adherence, indicating that sleep might influence dietary quality. Poor sleep is known to increase cravings for high-energy foods, weaken impulse control, and reduce motivation for healthy habits, all of which can hinder adherence to the MD [101]. In our study, parents’ higher levels of sleep quality were positively associated with better adherence to MD, which in turn significantly predicted children’s adherence to MD. This indirect link, potentially where parental sleep quality impacts parental MD adherence, which subsequently affects child MD adherence, requires further examination through longitudinal studies (to establish potential causalities).

Overall, our findings suggest that children’s adherence to the MD is shaped by multiple factors, including parental-sociodemographic factors, lifestyle behaviors, and dietary habits, thereby expanding the existing body of research on parental determinants of children’s MD adherence. Therefore, strengthening parental adherence to the MD, promoting structured meal routines, and reducing the availability of sugar-sweetened beverages at home may support healthier eating patterns in children. Interventions delivered through schools, community programs, and primary healthcare settings could involve parents directly, as their own habits strongly influence child outcomes. Given the declining adherence to the MD among youth in Mediterranean countries, such targeted, family-centered strategies are especially important for preserving traditional dietary patterns and preventing NCDs.

#### 4.1.1. Future Perspectives

The findings have important implications for future research and public health practice, highlighting the family unit as a key target for intervention. The strong associations between parental Mediterranean Diet (MD) adherence, structured meal routines, and children’s dietary patterns support the development of family-based interventions that address parental and child behaviors simultaneously. Previous research suggests that such approaches are effective, particularly when they combine parental nutrition education, practical skill-building, and behavioral strategies to establish consistent meal routines and reduce sweetened beverage availability in the home [23,37,102]. School-based programs that actively involve parents represent a feasible and scalable delivery setting [103], while longitudinal intervention studies are needed to assess sustainability and clarify causal pathways. The potential mediating role of parental sleep quality also warrants prospective investigation [6] as improving sleep health may represent an additional, underexplored lever for promoting healthier dietary patterns in children. Future research should prioritize longitudinal and interventional designs to confirm the observed associations and clarify causal pathways. The use of objective measures, investigation of family-level mechanisms, inclusion of more diverse populations, and evaluation of family-based, particularly school-based, interventions will be essential for developing effective, scalable strategies to improve Mediterranean Diet adherence among children and adolescents. Future studies could also explore potential interaction effects (e.g., education and diet) and stratified analyses (e.g., by children’s age group) to better identify subpopulations that may benefit most from targeted interventions. Finally, consideration of additional confounders such as family income, the food environment, time available for food preparation, and food culture would strengthen the analytical framework of future studies.

#### 4.1.2. Strengths and Limitations

To our knowledge, this is the first study in Greece to comprehensively examine how a wide range of parental lifestyle behaviors, including physical activity levels, sleep quality, daily dietary routines, and nutrition literacy, relate to children’s adherence to the MD. A major strength of this study is the inclusion of a large and diverse sample of parent–child dyads from Crete, enabling robust examination of multiple parental determinants of children’s adherence to the MD. However, certain limitations must be considered. Firstly, the cross-sectional design prevents determining cause-and-effect relationships, as observed associations could be due to reverse causation or shared factors like socioeconomic status. Secondly, the use of convenience sampling through online and school networks might have led to a biased selection of participants, over-representing health-conscious and highly educated parents. This could limit the generalizability of the results, especially in more advantaged groups, and potentially inflate the strength of observed associations. Furthermore, the limited variation in parental physical activity and nutrition literacy also restricts generalizability. The study’s focus on Crete, a region with a unique diet, and its reliance on predominantly urban, highly educated, and maternal respondents further reduces generalization. Furthermore, although socioeconomic factors were considered, the absence of household income data leaves open the possibility of residual socioeconomic confounding. Therefore, future studies could focus on other regions of Greece and other parts of Europe with a less homogenous sample, including participants with different socioeconomic statuses, a major factor in MD adherence and childhood obesity. For example, higher obesity and comorbidity rates in European high-income countries and poorer adherence to healthy diets (including MD) were more prevalent in children populations from minority ethnicities, which necessitates more targeted nutritional and associated lifestyle interventions and studies [104]. Lastly, using a single parent respondent for all data may introduce biases related to shared methods and social desirability and may not fully represent the influence of the entire household or fathers. Consistent with previous research from Greece [105], the majority of participants in our study were mothers rather than fathers. While this pattern reflects typical parental engagement in child-related research, the overrepresentation of mothers may limit the generalizability of findings regarding paternal influences on children’s dietary behaviors. Studies conducted in similar European communities but with lower socioeconomic status (e.g., minority ethnic groups) have shown higher participation rates from fathers than mothers [106].

## 5. Conclusions

In conclusion, our findings indicate that children’s adherence to the MD is linked to a range of parental demographic, lifestyle, and dietary factors. Specifically, younger parental age, higher educational attainment, lower BMI, urban residence, and strong parental adherence to the MD were all positively associated with more favorable dietary behaviors in children. Parental meal routines and healthier beverage choices also emerged as important contributors, while parental intake of sweetened beverages and prolonged weekend screen time were linked to poorer adherence. These findings underscore the central role of the family environment in shaping children’s nutritional behaviors and highlight opportunities for targeted, family-based interventions to promote healthier dietary patterns. Supporting parents in adopting consistent, health-promoting routines may be a key strategy in improving children’s adherence to MD and, ultimately, in reducing the risk of NCDs.

## Figures and Tables

**Table 1:** Baseline main characteristics of the 760 participants according to the MD Quality Index in Children and Adolescents (KIDMED).

		MD ^a^ Quality Index Score			
Parental Characteristics	Total (N = 760)	Poor ≤3 (N = 139)	Average 4–7 (N = 378)	Optimal 8–12 (N = 243)	*p*-Value
Demographics					
**Gender: female**	639 (84%)	111 (80%)	306 (81%)	222 (91%)	**<0.001**
**Age ≥ 45 years**	279 (37%)	73 (53%)	142 (38%)	64 (26%)	**<0.001**
**BMI ^b^** (kg/m^2^)	26.05 ± 5.15	27.91 ± 6.03	26.33 ± 5.06	24.55 ± 4.31	**<0.001**
BMI ≥ 30 kg/m^2^	149 (20%)	38 (28%)	83 (22%)	28 (12%)	**<0.001**
**Area of Residence**					
Rural	202 (27%)	58 (42%)	85 (23%)	59 (24%)	**<0.001**
Urban	558 (73%)	81 (58%)	293 (78%)	184 (76%)	
**Married**	689 (91%)	127 (91%)	340 (89%)	222 (91%)	0.452
**Working**	641 (84%)	124 (89%)	314 (83%)	203 (83%)	0.280
**Educational Level**					
Secondary Education	149 (20%)	36 (26%)	73 (19%)	40 (16%)	0.081
Tertiary Education	399 (52%)	73 (52%)	203 (54%)	123 (51%)	
Higher Education	212 (28%)	30 (22%)	102 (27%)	80 (33%)	
**Smoking status**					
Non-smokers	421 (55%)	82 (59%)	203 (54%)	136 (56%)	0.549
Current/Ex-smokers	339 (45%)	57 (41%)	175 (46%)	107 (44%)	

^a^ MD: Mediterranean diet, ^b^ BMI: body mass index.

**Table 2:** Baseline questionnaire scores and classifications of the 760 participants according to the MD Quality Index in Children and Adolescents (KIDMED).

		MD ^a^ Quality Index Score			
Parental Questionnaires	Total (N = 760)	Poor ≤3 (N = 139)	Average 4–7 (N = 378)	Optimal 8–12 (N = 243)	*p*-Value
**MD Adherence Screener (MEDAS ^b^)**—total score	8.26 ± 1.85	7.31 ± 1.79	8.03 ± 1.69	9.15 ± 1.74	**<0.001**
Poor (1–4)	51 (6%)	21 (15%)	25 (7%)	5 (2%)	**<0.001**
Average (5–7)	521 (69%)	105 (76%)	285 (75%)	131 (54%)	
Good (8–14)	188 (25%)	13 (9%)	68 (18%)	107 (52%)	
**International Physical Activity Questionnaire (IPAQ ^c^)**					
Low/Moderate	190 (25%)	39 (28%)	91 (24%)	63 (26%)	0.790
High	570 (75%)	100 (72%)	287 (76%)	183 (75%)	
**Pittsburgh Sleep Quality Index (PSQI ^d^)** total score	5.92 ± 2.85	6.04 ± 2.94	6.01 ± 2.88	5.73 ± 2.75	0.435
Poor Sleep Quality (PSQI ≥ 5)	509 (67%)	95 (68%)	257 (69%)	157 (66%)	0.596
**Nutrition Literacy Scale (NLS ^e^)**	25 (24, 27)	25 (23, 27)	26 (24, 27)	25 (24, 27)	0.522
Inadequate (<8)	0	0	0	0	0.569
Marginal (8–15)	5 (1%)	0	3 (1%)	2 (1%)	
Adequate (>15)	755 (99%)	139 (100%)	375 (99%)	241 (99%)	

^a^ MD: Mediterranean diet, ^b^ MEDAS: Mediterranean Diet Adherence Screener, ^c^ IPAQ: International Physical Activity Questionnaire, ^d^ PSQI: Pittsburgh Sleep Quality Index, ^e^ NLS: Nutrition Literacy Scale; KIDMED: Mediterranean Diet Quality Index in Children and Adolescents.

**Table 3:** Comparison of the percentage distribution of nutrition habits among the 760 participants according to the MD Quality Index in Children and Adolescents (KIDMED).

		MD ^a^ Quality Index Score			
Parental Behaviors	Total (N = 760)	Poor ≤3 (N = 139)	Average 4–7 (N = 378)	Optimal 8–12 (N = 243)	*p*-Value
Water intake at least 5 times per day	387 (51%)	61 (44%)	191 (51%)	135 (56%)	0.088
Fresh juice at least one portion/week	613 (81%)	99 (71%)	307 (81%)	207 (85%)	**0.004**
Other juices at least one portion/week	264 (35%)	51 (37%)	131 (35%)	82 (34%)	0.843
Beverages with sweeteners at least one portion/week	325 (43%)	68 (49%)	174 (46%)	83 (34%)	**0.004**
Beverages with sugar at least one portion/week	251 (33%)	57 (41%)	119 (32%)	75 (31%)	0.085
Breakfast daily	427 (56%)	50 (36%)	202 (53%)	175 (72%)	**<0.001**
Mid-morning snack daily	253 (33%)	30 (21%)	116 (31%)	107 (44%)	**0.001**
Mid-afternoon snack daily	240 (32%)	23 (17%)	114 (30%)	103 (42%)	**<0.001**
Snack before sleep	115 (15%)	12 (9%)	59(16%)	44 (18%)	**0.043**
Screen time ≥ 3 h daily in non-working hours on weekdays	211 (28%)	46 (34%)	107 (29%)	58 (24%)	0.137
Screen time ≥ 3 h daily in non-working hours on weekends	295 (40%)	68 (50%)	147 (39%)	80 (34%)	**0.008**

^a^ MD: Mediterranean diet.

**Table 4:** Adjusted associations between parental characteristics, lifestyle, and dietary behaviors (continuous scales) and KIDMED score, estimated by linear regression models.

Variables	KIDMED ^a^ Score (Range 0–12)	
	Beta (95%CI ^b^)	*p*-Value
Child’s age	−0.135 (−0.180, −0.091)	**<0.001**
Gender (females vs. males)	0.034 (−0.461, 0.529)	0.893
Age ≥ 45 years	−0.987 (−1.349, −0.625)	**<0.001**
BMI ^c^	−0.072 (−0.107, −0.037)	**<0.001**
Area of residence (urban vs. rural)	0.544 (0.163, 0.925)	**0.005**
Higher education	0.493 (0.096, 0.871)	**0.014**
Siblings	−0.486 (−0.988, 0.016)	**0.056**
MD ^d^ Adherence Screener (MEDAS ^e^)—total score	0.493 (0.401, 0.586)	**<0.001**
PA ^f^ classification	0.306 (−0.317, 0.928)	0.335
PSQI ^g^ total score	−0.016 (−0.080, 0.048)	0.622
Nutrition Literacy Scale (NLS ^h^)	−0.022 (−0.085, 0.041)	0.487
Water intake at least 5 times per day	0.482 (0.125, 0.838)	**0.008**
Fresh juice at least one/week	0.716 (0.260, 1.172)	**0.002**
Other juices at least one/week	−0.050 (−0.429, 0.329)	0.797
Beverages with sweeteners at least once/week	−0.497 (−0.863, −0.131)	**0.008**
Beverages with sugar at least once/week	−0.390 (−0.774, −0.005)	**0.047**
Breakfast daily	1.294 (0.917, 1.670)	**<0.001**
Mid-morning snack daily	0.810 (0.432, 1.189)	**<0.001**
Mid-afternoon snack daily	1.022 (0.638, 1.406)	**<0.001**
Snack before sleep	0.493 (−0.004, 0.990)	0.052
Screen time ≥ 3 h daily in non-working hours on weekdays	−0.196 (−0.598, 0.205)	0.338
Screen time ≥ 3 h daily in non-working hours on weekends	−0.383 (−0.755, −0.010)	**0.044**

^a^ KIDMED: Mediterranean Diet Quality Index in Children and Adolescents, ^b^ CI: Confidence Interval, ^c^ BMI: Body Mass Index, ^d^ MD: Mediterranean diet, ^e^ MEDAS: Mediterranean Diet Adherence Screener, ^f^ PA: Physical Activity, ^g^ PSQI: Pittsburgh Sleep Quality Index, ^h^ NLS: Nutrition Literacy Scale.

## Data Availability

The data presented in this study is available upon reasonable request from the corresponding author due to privacy and ethical restrictions.

## Supplementary Materials

The following supporting information can be downloaded at https://www.mdpi.com/article/10.3390/nu18101576/s1, Table S1: STROBE checklist.
